# Towards Optimal Sensor Placement for Cybersecurity: An Extensible Model for Defensive Cybersecurity Sensor Placement Evaluation

**DOI:** 10.3390/s25196022

**Published:** 2025-10-01

**Authors:** Neal Wagner, Suresh K. Damodaran, Michael Reavey

**Affiliations:** 1 The MITRE Corporation, Bedford, MA 01730, USA; 2 Department of Computer Science and Information Systems, University of North Alabama, Florence, AL 35632, USA

**Keywords:** cybersecurity, cyber sensor, optimal sensor placement, mathematical modeling, risk assessment, cyber threat modeling

## Abstract

Optimal sensor placement (OSP) is concerned with determining a configuration for a collection of sensors, including sensor type, number, and location, that yields the best evaluation according to a predefined measure of efficacy. Central to the OSP problem is the need for a method to evaluate candidate sensor configurations. Despite the wide use of cybersecurity sensors for the protection of network systems against cyber attacks, there is limited research focused on OSP for defensive cybersecurity, and limited research on evaluation methods for cybersecurity sensor configurations that consider both the sensor data source locations and the sensor analytics/rules used. This paper seeks to address these gaps by providing an extensible mathematical model for the evaluation of cybersecurity sensor configurations, including sensor data source locations and analytics, meant to defend against cyber attacks. We demonstrate model usage via a case study on a representative network system subject to multi-step attacks that employ real cyber attack techniques recorded in the MITRE ATT&CK knowledge base and protected by a configuration of defensive cybersecurity sensors. The proposed model supports the potential for adaptation of techniques and methods developed for OSP in other problem domains than the cybersecurity domain.

## 1. Introduction

The optimal sensor placement (OSP) problem is focused on determining the best configuration of sensors for a given system and monitoring task. A sensor placement configuration specifies the pertinent characteristics of a collection of sensors and includes determination of the types of sensors used (i.e., the analytics or rules used to detect attack), the number of sensors of each type, their locations, as well as other relevant aspects such as data transfer, storage, and processing techniques [1]. For any OSP application, a primary concern is how to evaluate a candidate sensor placement configuration for efficacy. Depending on the problem domain, monitoring efficacy may be a multi-objective measure that incorporates competing aspects of monitoring performance and cost, among others [1]. OSP for physical systems and processes has long been and continues to be an active research area spanning many problem domains. Some examples include [2,3,4,5,6,7,8,9,10,11]. A prevalent concern for network systems, including cyber–physical systems, is the threat of cyber attack, and thus defensive cybersecurity sensors are commonly employed to monitor and detect such attacks [12,13,14].

Despite the wide use of cybersecurity sensors for the protection of network systems, there is limited research focused on OSP for defensive cybersecurity, and limited research on evaluation methods for cybersecurity sensor configurations. The few instances of cybersecurity OSP studies that exist have focused on either data source locations or sensor analytics used, but none have considered the full cybersecurity OSP problem, which entails consideration of both data source locations and sensor analytics simultaneously, as is recognized in [15]. Existing Security Information and Event Management (SIEM) solutions, such as Elasticsearch [16] and Splunk [17], aggregate and store event logs from all analytics and locations implemented, but do not address the problem of how to decide where analytics should be placed or which analytics should be used.

To understand the importance of addressing both where sensors are placed and which analytics should be deployed at which locations, consider the findings from the 2025 Data Breach Investigations Report published by Verizon [18]. The report investigates 12,195 data breaches across multiple sectors of business and government. From the report, 20% of data breaches exploited vulnerabilities on services and edge devices, while another 16% were initiated by phishing attacks. Due to the large number of services and software available and the continuous stream of newly discovered vulnerabilities and patch updates, it is difficult for defenders to ensure that all services and edge devices are always fully patched. Furthermore, as discussed in the report, some attacks use zero-day exploits that even fully patched systems cannot prevent. Thus, incorporating effective sensor-based defenses is critical for network system protection. However, the complexity of modern network environments means that deployment of sensor-based defensive measures comes at a cost: more active sensors and monitoring cause more data processing, analysis, and storage requirements. Due to this cost, it is not feasible for defenders to monitor all possible locations with all available analytics. Therefore, defenders must judiciously decide which detection analytics should be deployed at which system locations to best counterattack threats of concern and keep resource costs within acceptable limits.

This paper aims to address this gap by proposing an extensible mathematical model for defensive cybersecurity sensor placement evaluation that considers the full version of the cybersecurity OSP problem, including both sensor data source locations and the sensor analytics/rules used. The proposed model enables the potential for adaptation of techniques and methods developed for OSP in other problem domains to the cybersecurity domain. We demonstrate model usage via a case study on a representative network system protected by a configuration of defensive cybersecurity sensors under multi-step attacks that employ techniques observed in real cyber breach incidents recorded in the MITRE ATT&CK knowledge base [19] against known vulnerabilities from the National Vulnerability Database (NVD) [20]. The contributions of this study are as follows.

- A novel and detailed problem formulation of the defensive cybersecurity optimal sensor placement problem.
- A novel, extensible mathematical model for quantitative evaluation of defensive cybersecurity sensor configurations to protect against cyber attack, including capture of both sensor data source locations and sensor analytics/rules used, the combination of which has never before been explored.
- A detailed case study demonstrating model usage for a representative network system protected by a configuration of defensive cybersecurity sensors and under the threat of multi-step attacks that employ real cyber attack techniques taken from the MITRE ATT&CK knowledge base against known vulnerabilities recorded in the NVD.
- A discussion on how the model can be extended and used to support future OSP research efforts for defensive cybersecurity.

The rest of this paper is organized as follows: Section 2 discusses related work, Section 3 describes the defensive cybersecurity optimal sensor placement problem and provides a formalized and detailed mathematical problem formulation, Section 4 details the proposed mathematical model for cybersecurity sensor placement evaluation, Section 5 gives the case study used for experimentation and model demonstration, Section 6 discusses experiments conducted on the case study and presents results and analysis, Section 7 provides a discussion of practical considerations for applying the model to real-world systems and outlines several directions of work that can serve to support future defensive cybersecurity research efforts, and Section 8 concludes.

## 2. Related Work

OSP for physical systems and processes is an ongoing area of research that has been active for decades across several problem domains. Some examples include structural monitoring [1,4,9,10,21], environmental and agricultural monitoring [22,23,24,25,26], water systems [2,5,27], industrial and manufacturing systems [3,11,28,29,30], transportation systems [24,31], robotics and UAVs [7,8,32,33], location and position tracking [6,34], and human movement analysis [35], among others.

In the cybersecurity domain, sensors and monitoring research are largely focused on techniques, methods, and analytics for cyber attack detection. Several studies propose novel detection techniques specific to a particular problem domain. In [36,37], novel computational intelligence and machine learning techniques are developed to detect cyber attacks on healthcare data systems. Ref. [38] proposes fog computing techniques coupled with machine learning methods for efficient detection of cyber attacks on water distribution systems. In [39], several machine learning techniques are examined for their performance in detecting cyber spoofing attacks on global navigation satellite systems (GNSSs). Ref. [40] develops a method for detecting puppet attacks on fingerprint scanner devices used for multi-factor authentication, while [41] provides a process for detecting cyber attacks on DC micro-grid sensors on electric vehicles. Ref. [13] provides a survey of current techniques designed to detect cyber attacks on smart grids , while [12,14] provide reviews on recent machine learning methods for cyber attack detection in IoT systems and cyber–physical systems, respectively.

Other cybersecurity sensor studies propose techniques that are designed to detect specific types of cyber attacks. In [42,43], detection of cyber-based sensor deception attacks is the focus. Both studies propose a Discrete Event Simulation (DES) model for estimating the state of a given cyber–physical system under a deception attack. Ref. [44] provides a novel spam detection technique that leverages a long short-term memory (LSTM) neural network model to capture more semantic information than can be captured by traditional network models.

Another active stream of cybersecurity sensor research is focused on methods for the detection of cyber attacks on partially or fully autonomous and connected vehicles. In [45], a Bayesian estimation technique is proposed for anomaly detection of cyber threats on connected cars. Ref. [46] provides a detection method for both cyber attack detection and radar sensor health monitoring in semi-autonomous adaptive cruise control (SA-ACC) connected vehicles. In [47], an analytical framework based on an LSTM neural network and a parametric Gaussian process model is proposed to detect compromised connected vehicles in a transportation network using only stationary sensor data. Another relevant work explored a non-parametric Bayesian framework that addresses detection of attacks on cyber–physical systems such as aircraft and spacecrafts using a sticky Hierarchical Dirichlet Process Hidden Markov Model (sHDP-HMM) [48].

Yet another area of cybersecurity sensor research focuses on Intrusion Detection System (IDS) placement, that is, the data source location on which to place attack detectors. In [49], a greedy algorithm based on static analysis of attack graphs for detector data source location placement is used. Ref. [50] provides qualitative strategies for detector data source placement in Network-based Intrusion Detection Systems (NIDSs). In [51,52], detector data source placements designed to mitigate botnets are examined. In [15], cybersecurity sensor placements that vary the placement of sensor analytics rather than detector data source locations (which are assumed to be fixed) are considered. A placement of sensor analytics (referred to as IDS rule deployments) is evaluated as to whether or not it can detect a given attack threat, but not how likely it is to detect the threat.

Although OSP for cyber–physical systems continues to be an active research area, studies have examined sensor configurations meant to detect unsafe conditions rather than cyber attacks. Cybersecurity sensor and monitoring research, while also an active area, has focused primarily on algorithms and techniques to detect cyber attacks or, in a few instances, detector data source location placement or sensor analytics used. However, there is a gap in the research on the full version of the cybersecurity optimal sensor placement problem, which is concerned with determining an optimal defensive sensor placement that considers both detector data source locations and sensor analytics used [15].

This paper aims to address the full version of the cybersecurity optimal sensor placement problem, that is, to determine an optimal defensive cybersecurity sensor placement that assigns detector data source locations and analytics/rules used for detection. With this aim, this paper proposes an extensible mathematical model for defensive cybersecurity sensor placement evaluation. The proposed model captures adversarial dynamics between attack threats, as represented by realistic multi-step cyber attacks employing real attack techniques taken from the MITRE ATT&CK knowledge base [19], and cybersecurity sensor and monitoring defense, including sensor placements with varying detector data source locations and detector analytics. Defensive sensor placements are quantitatively evaluated for their risk and consider not only whether or not a given sensor placement *can* detect a given attack threat, but also *how likely* it will be able to detect the threat. The proposed model supports the application of advances in OSP research from other problem domains to be adapted to the cybersecurity domain.

## 3. The Cybersecurity Sensor Placement Problem

This section aims to describe and formalize the cybersecurity optimal sensor placement (COSP) problem. To provide the proper background and context, we first introduce and discuss preliminary concepts necessary to understand the COSP problem (Section 3.1). We then provide foundational definitions (Section 3.2) and a mathematical problem formulation (Section 3.3).

### 3.1. Preliminary Concepts

Cyber attacks are often described in terms of the Tactics, Techniques, and Procedures (TTPs) that a threat actor employs to execute malicious actions on a network system. An attack TTP describes both what the threat actor is doing and how they are doing it [19,53,54]. Cyber attacks are detected primarily through the analysis of event logs generated by system components, such as devices, workstations, and servers, as they operate. System component event logs are collected from the networks in which they reside. For example, System Monitor (Sysmon) is a Windows system service and device driver that runs in a protected process, persists across system reboots, and records system activity [55]. Sysmon logs track system processes, network connections, and file changes. Packet capture (pcap) logs record network packets in transit over a network and can be collected via monitoring software such as libpcap or npcap for Unix or Windows systems, respectively [56,57]. Network pcap logs are typically collected from a spanport of a network switch. Security Information and Event Management (SIEM) systems are widely used to collect event logs and query them using domain-specific languages. For example, Elasticsearch is a widely deployed SIEM that is used to aggregate event logs from various data sources for the querying of anomalous events [58]. As the number of system components in computer networks increases, the data collected from them increases accordingly. The enormous volume of data that can be collected from modern networks poses a problem for network security administrators due to the data processing and analysis required to monitor, detect, and respond to cyber attacks [59].

In safety-critical environments, there is an increasing desire for detecting attacks in real time [14,60]. In such environments, one option is to collect the event data in a centrally located SIEM and then run real-time queries in the SIEM. Another option is to perform anomalous event detection using one or more IDS closer to where event logs are generated. The advantage of the latter option is that the data rate and the volume are lower, and, when low size, weight, and power (SWaP) detection tools are used, anomalous events can be detected relatively faster than can be detected via queries executed on a centralized SIEM [61]. In a complex safety-critical system that contains numerous hardware and software components, it can be expensive to extract operational event logs for the purpose of detecting cyber attacks and reducing cybersecurity risk. Even when operational event logs are extracted from every possible hardware and software component of a given system, irrespective of whether an SIEM or another detection closer to the origin of the event logs is used, there is a cost associated with how many analytical queries can be run simultaneously on a cyber attack detection tool [62]. This limitation demands that an optimal number of event data sources must be chosen at any moment for conducting analytical queries in order to maximize cybersecurity defensive posture and keep associated costs within acceptable limits.

The COSP problem is further complicated by the need to select the type of analytics used to detect cyber attacks when monitoring event logs from a given system component. Generally, there exist two types of cyber attack detection analytics: signature-based analytics and behavior-based or anomaly-based analytics [61,63]. Signature-based analytics are designed to detect specific known cyber attack techniques using pattern-matching methods that identify previously observed malicious patterns and small variants of those patterns [64]. Anomaly-based analytics, rather than targeting previously seen attacks, seek to identify malicious anomalous behavior that deviates from the normal, non-malicious behavior present on a given network system [61,65].

Both types of analytics have associated advantages and disadvantages. Signature-based analytics, when properly implemented, can provide quick and accurate detection of targeted attack techniques. However, existing analytics must constantly be updated as malware signatures change over time, and new analytics must continually be added to capture new and evolving attack threats. This poses a significant burden on network administrators both in terms of time and expertise. Additionally, analytics that are improperly maintained and updated can lead to high false positive and false negative rates [66]. Anomaly-based analytics have the advantage of being able to detect both known and *unknown* (zero-day) attacks [67]. However, anomaly-based analytics require training on a relatively large dataset of representative non-malicious behavior to be effective and must be continually retrained as applications evolve and usage patterns change [68]. Both signature-based and anomaly-based analytics, even when properly maintained and updated, may still be subject to non-trivial false positive and false negative rates.

### 3.2. Definitions

This section provides definitions for several key concepts of the COSP problem.

**Definition 1** (Cyber Attack Trace)**.**
*A cyber attack trace characterizes a single sequence of actions executed by a threat actor on a network system to achieve a malicious goal. A cyber attack trace is represented as a tuple, 〈g,AS〉, in which g represents the attacker goal and $\mathrm{AS}=as_{1},as_{2},\ldots ,as_{n}$ is a sequence of n≥1 attack steps, $as_{i}$ representing the $i^{\mathrm{th}}$ attack step of AS, executed on system components to attain g.*

It is important to note that an adversary may employ multiple different cyber attack traces when executing a cyber attack to improve their chance of success. Additionally, a single cyber attack trace may include attack steps that may be executed in parallel. When this is the case, the order of attack steps in the sequence is generated by the chronological order of attack step completions. Note that there could be multiple equivalent attack traces if the chronological order of some of the attack steps does not alter the reachability to the final attack step in the sequence $as_{n}$.

**Definition 2** (Cyber Attack Step)**.**
*A cyber attack step characterizes a single action taken by the attacker on a system component to advance the attack’s progress toward its goal. A cyber attack step is represented as tuple,*

$$as_{i}=\langle act,cmp\in CMP,active\rangle$$

*where act is the attacker action, cmp is the system component upon which the action is taken, CMP is the set of hardware and software components that make up a given network system, active is a boolean representing whether it is possible to execute act on cmp (i.e., whether the attacker has gained the appropriate privileges to allow the action to be executed on the component), and $as_{i}$ represents a single attack step in the sequence of attack steps AS present in a given cyber attack trace. Furthermore, $result\left(as_{i}\right)$ is a boolean function that captures the outcome of step $as_{i}$, which if true, implies that the active element of the next attack step in the sequence is set to 1, that is $result\left(as_{i}\right)=1\Rightarrow activate\left(as_{i+1}\right)$ where activate is a function that sets the value of the active element for a given attack step to 1.*

Note that it is possible for multiple different attack steps to be executed on a single component. For example, an attacker may execute one attack step on a particular component that logs keyboard inputs to steal credentials, and then, in a following attack step, use those stolen credentials to escalate privileges on the very same component.

**Definition 3** (Cybersecurity Data Source)**.**
*A cybersecurity data source is an event log that is collected about an individual component of a network system and is represented as a tuple, $\langle el_{type},cmp\rangle ,cmp\in CMP$, in which $el_{type}$ represents an event log type, CMP is the set of hardware and software components that make up a given network system, and cmp represents a single component in CMP.*

Note that it is possible for the same type of event log to be collected for potentially multiple individual components of a given network system.

**Definition 4** (Cybersecurity Analytic)**.**
*A cybersecurity analytic is a machine-executable analytic, or method of logical analysis, that is used to analyze event logs from one or more cybersecurity data sources for the purpose of detecting one or more cyber attack steps.*

**Definition 5** (Cybersecurity Sensor)**.**
*A cybersecurity sensor is the combination of one or more cybersecurity data sources together with a cybersecurity analytic for the purpose of detecting one or more cyber attack steps and is represented as a tuple, $\langle DS_{sn},anl\rangle$, where $DS_{sn}$ is the set of data sources that the sensor monitors $DS_{sn}\neq \emptyset$ and anl is the analytic used to analyze the data source(s) of $DS_{sn}$.*

Note that a cybersecurity sensor may monitor a single data source for malicious behavior, or it may monitor two or more data sources and correlate events across these data sources in order to detect malicious behavior.

**Definition 6** (Cybersecurity Sensor Placement)**.**
*A cybersecurity sensor placement is a configuration of one or more cybersecurity sensors that are enabled to monitor event logs from one or more data sources originating from one or more components of the network system for the purpose of detecting one or more cyber attack steps.*

As given in Definition 5, a cybersecurity sensor needs to monitor event log(s) from one or more data sources in order for its analytics to detect anomalous behavior. We do not make any assumption as to where the sensor is physically deployed. In some defensive cybersecurity operations in which data sources are collected and processed centrally, sensors are often physically deployed at the point of centralized computation. Another common alternative is to place sensors physically closer to the location of the data sources being monitored. A hybrid approach may also be used, in which some sensors are placed physically close to the components from which event logs are generated, while other sensors are located centrally. In this context, what we mean by *cybersecurity sensor placement* is not the physical deployment location of sensors, but rather which data source(s) are being monitored and which analytics are used to monitor them for anomalous behavior, irrespective of a sensor’s physical location.

Further, there is no assumption as to the monitoring approach used by a given cybersecurity sensor placement. Generally, there exist two monitoring approaches: a streaming analytical approach and a batch-oriented approach. A streaming analytical approach monitors data source event logs as soon as they are available to the sensor. Some examples of this approach include analytical tools Apache Flink [69] and MITRE Cyber SEAL [70]. In a batch-oriented approach, sensor data is collected in time-ordered groups or batches, and a sensor’s analytics operate on a batch of collected data when it becomes available. Examples of this approach, which is sometimes referred to as the Big Data approach, include commercial tools such as Splunk [17] and Elastic [16]. We do not make any assumption as to the monitoring approach used, and only assume that threat detection by sensors occurs in a relatively timely fashion to enable administrators to enact appropriate defensive measures to effectively defend monitored system components.

For the remainder of this paper, for convenience, we interchangeably refer to a cyber attack trace as simply an “attack trace”, a cyber attack step as simply an “attack step”, a cybersecurity data source as a “data source”, a cybersecurity analytic as an “analytic”, a cybersecurity sensor as a “sensor” or a “cyber sensor”, and a cybersecurity sensor placement as a “sensor placement” or a “sensor placement configuration”. We provide detailed examples of each of these concepts in Section 5.

### 3.3. Problem Formulation

We formulate the COSP problem as an optimization problem in which, given a set of cyber sensors SN that may be enabled or disabled to monitor and detect attack steps from a given set of attack traces T, we wish to decide a sensor placement sp⊂SN to minimize the objective function

(1) $$Risk=r_{T}\left(sp\right)=1-\left[\prod_{tr\in T}1-r_{tr}\left(sp\right)\right],$$

subject to the constraints

(2) $$\begin{array}{cc} Cost & =c\left(sp\right)\leq c_{max},\\ Risk & =r_{T}\left(sp\right)\leq r_{max}, \end{array}$$

where tr is a single attack trace in T and varies across all traces of T, $r_{tr}$ is a function that computes the inherent risk that tr places on the system given the defensive cybersecurity sensor placement sp and returns a value in [0,1] representing the probability that tr attains its malicious goal, *c* is a function that computes the cost associated with using sp, $c_{max}$ is a constant representing the maximum acceptable cost, $r_{T}$ is a function that computes the overall risk associated with the set of attack traces T, and $r_{max}$ is a constant representing the maximum acceptable risk.

If it is feasible to use all available sensors, that is, $c\left(sp=\mathrm{SN}\right)\leq c_{max}$, then the problem becomes a multi-objective optimization problem:

(3) $$\begin{array}{cc} \mathrm{minimize}\;\; & \left\{r_{T}\left(sp\right),c\left(sp\right)\right\}\\ \mathrm{subject}\;\mathrm{to}\;\; & r_{T}\left(sp\right)\leq r_{max}, \end{array}$$

where $r_{T}$ and $c\left(sp\right)$ are the risk and cost functions given in Equations (1) and (2).

## 4. An Extensible Mathematical Model for Cybersecurity Sensor Placement Risk Evaluation

Solving the COSP problem requires a method for evaluating the risk and cost functions given in Equations (1) and (2). This section describes an extensible mathematical model for cybersecurity sensor placement risk evaluation. The proposed model aims to be a generalized model that enables quantitative comparison of the relative risk of two or more candidate sensor placements. Evaluating the cost of a given sensor placement is highly specific to the analytics, data sources, and computational processes used to monitor and detect cyber attacks, and we recognize modeling sensor placement costs as a direction for future work.

The proposed sensor placement risk evaluation model is intentionally general to allow for applicability across a wide variety of networks and cyber–physical environments and against a wide variety of cyber attack threats. The model is also designed for extensibility, so that it may be modified or enhanced to include details tailored to a specific cyber system and/or attack threat. For convenience, we refer to the cybersecurity sensor placement risk evaluation model interchangeably as the “sensor placement model” or simply “the model”.

The goal of a sensor placement configuration is to minimize the risk posed by one or more cyber attack traces. Risk is characterized by considering the probability of an undesired event (e.g., a successful cyber attack) and the damage that the event would cause [71]. Damage caused by a successful cyber attack is highly specific to the organizational mission that the network system supports, and thus, for the purposes of the proposed generalized model, we assume that any attack trace in the set of traces being evaluated (T of Equation (1)) represents a significant adverse impact to the system.

To model the risk that is incurred by a given collection of cyber threats, we consider the following aspects of a network system: (1) system components (both hardware and software) and their interconnections (either already existing or attacker generated), (2) component-level hardware and software vulnerabilities, (3) attack threats as captured by T, the given set of cyber attack traces for which a sensor placement is to be evaluated against, and (4) the sensor placement sp representing a configuration of defensive cybersecurity sensors to monitor and detect cyber attack. To promote extensibility, we use a modular modeling approach to capture details specific to individual system components, sensors, and traces as well as to capture dynamics that occur at different scales (e.g., component-level dynamics vs. network-level dynamics). Thus, the sensor placement model is divided into three sub-models: *the component vulnerability-to-threat sub-model*, *the sensor detection sub-model*, and *the cyber threat propagation sub-model*. The following sections describe these sub-models and discuss how they are combined to capture the full sensor placement model.

### 4.1. The Component Vulnerability-to-Threat Sub-Model

Vulnerabilities in a system component present exploit opportunities to an attacker who seeks to compromise the component and pivot to move laterally to other system components. The component vulnerability-to-threat sub-model captures the threat of successful compromise when a component with one or more known vulnerabilities is attacked. The sub-model characterizes devices with more vulnerabilities and/or more severe vulnerabilities as more likely to be compromised when attacked. We utilize the National Vulnerability Database (NVD) [20] to collect known vulnerabilities associated with a given system component, and to measure vulnerability severity, we leverage the Common Vulnerability Scoring System (CVSS) [72,73].

The number of vulnerabilities discovered has increased dramatically over the years [74], and this increase poses a significant risk to the secure usage of software-dependent components present in modern systems [75]. The vast and ever-changing vulnerability landscape, coupled with the large number of possible attacks [76], makes modeling and analysis of vulnerability-to-threat dynamics challenging. It is not tractable to model the details of every known vulnerability against every known attack. Thus, we aim to construct a generalized model to estimate the relative probability of compromise when a component with a given set of vulnerabilities is under attack. To this end, we employ the probabilistic model given by [77,78] and later used in a study analyzing the effectiveness of host-level cybersecurity defensive mitigations [79] to compute the probability of component compromise when an attack is initiated on a component with a given vulnerability profile (i.e., set of vulnerabilities).

(4) $$p_{compromise}\left(cmp\right)=1-\prod_{v\in V}\left(1-p_{compromise}\left(v\right)\right),$$

where *v* is a single vulnerability and varies over all known vulnerabilities *V* present on the component and $p_{compromise}\left(v\right)$ is the probability that an attack can successfully exploit *v* to compromise the component. $p_{compromise}\left(v\right)$ is computed as the normalized CVSS score by

(5) $$p_{compromise}\left(v\right)={\left(\frac{CVSS\left(v\right)}{10}\right)}^{2},$$

where $CVSS\left(v\right)$ is the CVSS score for *v* as listed in the NVD and $p_{compromise}\left(v\right)$ generates a value in [0.0,1.0].

The model given by Equations (4) and (5) assumes that the attack includes technique(s) to exploit all known vulnerabilities present on the component *V* and represents a worst-case scenario for system defense. Depending on the traces being evaluated and the vulnerabilities present on the component, a subset of vulnerabilities present on the component may not be relevant; that is, the attacker does not gain any advantage by exploiting them, or the attack traces being evaluated do not include attack steps that can exploit them. When this is the case in Equation (4), set *V* should include only *relevant* vulnerabilities, that is, those relevant to the traces being evaluated.

### 4.2. The Sensor Detection Sub-Model

The development of new and improved analytics, both signature-based and anomaly-based, is an active area of research [61,63], and thus, there is a very large number of possible analytics that can be used in a sensor placement. Additionally, as discussed in Section 3.1, there is an enormous number of possible data sources that can be monitored by analysis, and attack threats are constantly evolving. It is not tractable to model the details of every cyber sensor to detect all possible threats. Similar to the component vulnerability-to-threat sub-model, our goal is to construct a generalized model to estimate the detection probability of a sensor to detect an extent attack threat. Ideally, the detection probability for a given sensor to detect a given threat, in the form of an attack action that uses a given attack technique, may be estimated based on test results, data collected from red team exercises, or other analyses specific to the given sensor and attack technique considered. However, it is often not feasible to perform such tests/analyses for every relevant sensor and attack technique pairing, especially for larger-scale COSP problem instances.

For COSP problem instances in which such data is not available for all needed sensor and attack technique pairings, we propose the following generalized method for estimating a given signature-based sensor’s detection probability against a given attack step. The method makes use of publicly available sensor analytics data repositories, such as MITRE Cyber Analytics Repository [80], Sigma [81], ES SIEM [16], and Splunk [17], among others, that enumerate known analytics designed to detect attack techniques present in the MITRE ATT&CK knowledge base [19]. First, for each MITRE ATT&CK technique, record the total number of publicly available analytics designed to detect that technique. Once these totals have been recorded, the detection probability of a sensor monitoring an attack step that utilizes a given MITRE ATT&CK technique can be estimated by

(6) $$dp\left(s,as_{i},t_{j}\right)=min\left(\frac{|analy_{t_{j}}|}{|analy_{t}{|}_{max}},1.0\right),$$

where $t_{j}$ is the MITRE ATT&CK technique utilized by attack step $as_{i}$, $|analy_{t_{j}}|$ is the total number of public analytics designed to detect $t_{j}$, $|analy_{t}{|}_{max}$ represents a theoretical upper bound for total analytics available for any given attack technique, and $dp\left(s,as_{i},t_{j}\right)$ represents the estimated detection probability of sensor *s* to detect attack step $as_{i}$ that utilizes technique $t_{j}$. Intuitively, the model characterizes a sensor’s detection probability against an attack step that uses a given MITRE ATT&CK technique as relatively higher for attack techniques with many public detection analytics available compared to attack techniques with few available analytics. The theoretical upper bound $|analy_{t}{|}_{max}$ in Equation (6) can be specified by subject matter expertise, or it can be computed from the full distribution of total available analytics to individual MITRE ATT&CK techniques.

The sub-model given in Equation (6) captures a given sensor’s probability to detect a monitored attack step action when executed, that is, its true positive rate. The false positive rate, as well as the cost associated with handling false positives, are not modeled and are identified as a direction of future work.

As discussed in Section 3.1, anomaly-based sensors are not designed to detect a particular attack technique, but instead are designed to detect malicious behaviors that deviate from normal, non-malicious user behaviors. Thus, the generalized method described above for estimating the detection probabilities for signature-based sensors against specific attack techniques is not applicable. For anomaly-based sensors, ideally, test result data or other relevant analyses should be used to specify detection probability. When such data is not available, it is possible to estimate detection probability using a Bayesian approach in which the initial estimate is specified by prior belief and updated as new evidence becomes available [82]. Extension of the proposed sensor detection sub-model to incorporate performance estimation of anomaly-based sensors is identified as a direction of future work.

It is also important to note that the above generalized method for estimating a signature-based sensor’s detection probability (Equation (6)) is not meant to replace more precise methods that are geared specifically toward individual signature-based analytics and may vary widely depending on the analytic. Rather, the generalized method is intended as a data-driven and relatively non-resource-intensive estimation method when more precise methods, such as testing and/or analyses specific to each individual signature-based sensor, are too costly to execute.

### 4.3. The Cyber Threat Propagation Sub-Model

The cyber threat propagation sub-model captures an attack trace progressing through the system from its initial entry point; its intermediate steps over one or more components to its end point, at which point the attacker either attains its goal or is thwarted by extant defensive measures. The model also captures the impact of multiple attack traces, executed by one or more attackers, who wish to achieve potentially a variety of malicious goals. The model’s purpose is to estimate the security posture of a given system protected by a given defensive cybersecurity sensor placement against a given set of relevant attack traces. The model contains two sub-models. One is designed to capture a single attack trace through the system and compute its system impact. The other is designed to aggregate the effects of two or more traces on the system. The following describes these two sub-models.

#### 4.3.1. Single Attack Propagation Sub-Model

The single attack propagation sub-model captures the execution of a single attack trace as it progresses through the system towards its goal. The model is given by a directed acyclic graph $G=\left(N,E\right)$ in which *N* is a set of nodes representing individual attack steps and *E* is a set of directed edges representing an adjacency between successive attack steps where $E\subset \left(x,y\right)|\left(x,y\right)\in N^{2},x\neq y$. $x_{entry},x_{goal}\in N$ are two special nodes that represent the attack entry step, which is the first step in the sequence of attack steps of a given attack trace and marks the attacker’s entry into the system, and the attack goal step which, when successfully executed, allows the attacker to attain its intended goal.

**Figure 1:**
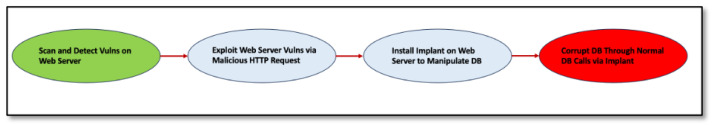
Directed graph representation of an example attack trace that exploits a web server to gain access to a database. The green and red colored nodes represent the attack entry and goal nodes of the graph, respectively.

Figure 1 shows the directed graph representation of an example attack trace whose goal is to corrupt a database that is accessible via a web server. From the figure, the attack entry step (green node) specifies the initial attacker action that scans the web server to detect exploitable vulnerabilities. The next attack steps include exploitation of detected web server vulnerabilities to gain escalated privileges and the installation of an implant on the web server that can be used to interact with the database (blue nodes). The attack goal step (red node) specifies the final attack step in which the DB is manipulated and corrupted via normal DB calls made through the installed implant. The attack steps of this example attack trace capture observed attacks described by MITRE ATT&CK techniques T1505, T1505.03, and T1057 [19].

The single attack propagation sub-model leverages the component-vulnerability-to-threat sub-model to capture the probability of success for individual attack steps of a given attack trace. Each attack step is associated with a system component and its relevant vulnerabilities, and the probability of compromise is precomputed by this model (Section 4.1). Additionally, the propagation model utilizes the sensor detection sub-model (Section 4.2) to capture the probability of attack detection when an attack step is executed on a system component that is being monitored by a given cyber sensor. We assume that an attack step detection implies successful defensive mitigation of the attack trace that utilizes it.

The propagation model employs Monte Carlo (MC) simulation to execute a set of probabilistic experiments on the graph model in which a single MC trial samples from the precomputed distributions for attack step success and attack detection associated with attack step nodes in the graph and records an outcome of TRUE if the evaluated attack trace successfully attains its intended goal, as signified by successful execution of the final attack goal step, and FALSE otherwise. The propagation model computes a random binary variable *v* that maps to the value of 1.0 when a recorded outcome is TRUE and a value of 0.0 when a recorded outcome is FALSE. The model outputs a vector $V=v_{1},\ldots ,v_{j}$ representing the set of outcomes generated by executing *j* MC trials on the model for a given attack trace and sensor placement. We define an experiment on the single attack propagation sub-model to be the execution of a set of MC trials on the model for a given parameter tuple, env=〈tr,sp〉, which specifies the experimental environment where tr and sp represent the attack trace and sensor placement employed, respectively. A given experiment generates the output vector $V_{env}$, which contains experiment outcomes corresponding to the model parameter tuple env. Algorithm 1 specifies the procedures executed by the single attack propagation sub-model.

In Algorithm 1, procedure SINGLE-ATTACK-PROPAGATION-TRIAL$\left(env\right)$ executes one MC trial of the single attack propagation sub-model for experimental environment env consisting of a single attack trace tr and sensor placement sp. This procedure computes the probability of attack step success for a single step of tr (line 16) as the probability that the attacked system component is successfully compromised *and* that no enabled sensor detects the attack step action. It then executes a random draw to decide the outcome, and if positive, activates the next attack step in the trace. If a negative outcome occurs for any attack step of the trace, the procedure returns a result of 0 indicating attack trace failure; otherwise, it returns a result of 1 indicating attack trace success (all steps succeeded). Procedure EXECUTE-SINGLE-ATTACK-PROPAGATION-TRIALS executes a set of individual trials for a given experimental environment env and returns a vector of trial outcomes $V_{env}$. Note that an attack step may not require exploitation of any vulnerability to successfully compromise a component (lines 6–7). This is the case when one or more previously executed successful attack steps gain the privileges necessary for the current attack step’s intended action. Such attack steps are often referred to as “living off the land” [83].
**Algorithm 1** Single attack propagation sub-model algorithm1: **procedure** Single-attack-propagation-trial(env=〈tr,sp〉)2:        n=|AS|▹ Size of attack sequence3:         $activate\left(as_{1}\right)$▹ Activate attack entry step4:        **for** i=1 to *n* **do**5:              $V=\mathrm{vulns}.\;\mathrm{present}\;\mathrm{on}\;as_{i}.cmp$▹ Set of vulns. on attacked component6:              **if** exploit of vulnerability not required **then**7:                    $p_{compromise}\left(as_{i}.cmp\right)=1$▹ Live-off-the-land exploit8:              **else if** V=∅ **then**9:                    $p_{compromise}\left(as_{i}.cmp\right)=0$10:              **else**11:                    Compute $p_{compromise}\left(as_{i}.cmp\right)$12:              **if** $applicable\left(s\in sp,as_{i}\right)$ **then**▹ Sensor enabled to detect attack step13:                    Compute $dp\left(s,as_{i},t_{j}\right)$▹ Detection probability14:              **else**15:                    $dp\left(s,as_{i},t_{j}\right)=0$16:              $\begin{array}{c} p_{result\left(as_{i}\right)}=p_{compromise}\left(as_{i}.cmp\right)\;\cdot \\ \;\left(1-dp\left(s,as_{i},t_{j}\right)\right) \end{array}$▹ Probability of attack step success17:              $rnd=random\left(0.0,1.0\right)$▹ Random draw18:              **if** $rnd<p_{result\left(as_{i}\right)}$ **then**19:                    **if** i<n **then**20:                           $activate\left(as_{i+1}\right)$▹ Activate next attack step21:                    **else**22:                           **return** 1▹ Attack attains goal23:              **else**24:                    **return** 0▹ Attack step failure, attack fails25: **procedure** Execute-single-attack-propagation-trials(num,env=〈tr,sp〉)26:       $V_{env}=\emptyset$▹ Initialize vector of trial results27:       **for** i=1 to num **do**28:             $result_{i}=\mathrm{Sin}\mathrm{gle}\text{-}\mathrm{attack}\text{-}\mathrm{propagation}\text{-}\mathrm{trial}\left(env\right)$29:             $append\left(V_{env},result_{i}\right)$30:       **return** $V_{env}$


#### 4.3.2. Multi-Attack Aggregation Sub-Model

The purpose of the multi-attack aggregation sub-model is to estimate the combined impact of a set of attack traces, each representing different attacks with potentially different malicious goals, on the system being evaluated. The model ingests results from multiple experiments run on the single attack propagation sub-model, where each experiment is specified by the parameter tuple $\langle num,env_{i}\rangle$ representing the number of MC trials to run and experimental environment, respectively, and generates the outcome vector $V_{env_{i}}$. The multi-attack aggregation sub-model computes the expected probability of attack impact (pai) for a given $V_{env_{i}}$ by

(7) $$pai\left(V_{env_{i}}\right)=\frac{\sum_{j=1}^{|V_{env_{i}}|}v_{j}}{|V_{env_{i}}|},$$

where $|V_{env_{i}}|$ is the size of vector $V_{env_{i}}$, $v_{j}$ represents outcome *j* from $V_{env_{i}}$, and $pai\left(V_{env_{i}}\right)$ represents the expected probability of attack impact for $V_{env_{i}}$. When a set of multiple attack traces is evaluated, the multi-attack aggregation sub-model generates a vector of attack impact computations in which elements represent individual traces evaluated. For example, if $T=\{tr_{1},tr_{2},tr_{3}\}$ is the set of attack traces being evaluated and $V_{T}=V_{env_{1}},V_{env_{2}},V_{env_{3}}$ is the vector of outcome vectors generated by experiments run on the single attack propagation sub-model for experimental environments $env_{1}=\langle tr_{1},sp\rangle$, $env_{2}=\langle tr_{2},sp\rangle$, $env_{3}=\langle tr_{3},sp\rangle$, respectively, where sensor placement sp remains constant over all experiments and each experiment is executed for a number of MC trials num, then the multi-attack aggregation sub-model generates a vector of expected probabilities of attack impact $pai_{T}=pai_{V_{env_{1}}},pai_{V_{env_{2}}},pai_{V_{env_{3}}}$, where individual elements correspond to computed probabilities of attack impact for traces $tr_{1},tr_{2},tr_{3}$, respectively, and sensor placement sp.

Additionally, the multi-attack aggregation sub-model computes the overall (cumulative) expected probability of attack impact due to any of the traces being evaluated by

(8) $$pai_{cumulative}\left(pai_{T}\right)=1-\left(\prod_{k=1}^{|pai_{T}|}1-pai_{T,k}\right),$$

where T is set of attack traces being evaluated, $pai_{T}$ is the vector of expected probabilities of attack impact computed for individual traces of T, $|pai_{T}|$ is the size of vector $pai_{T}$, *k* is an index variable over the indices of $pai_{T}$, $pai_{T,k}$ represents the individual element of $pai_{T}$ given at index *k*, and $pai_{cumulative}\left(pai_{T}\right)$ is the cumulative expected probability of attack impact due to any trace in T.

The multi-attack aggregation sub-model links all other sub-models of the sensor placement risk evaluation model and is used to compute the risk function of Equation (1), which represents the overall risk value associated with a given sensor placement assessment. Algorithm 2 specifies the procedures executed by the multi-attack aggregation sub-model.
**Algorithm 2** Multi-attack aggregation sub-model algorithm1: **procedure** Multi-attack-trace-pai(numOfTrials,env=〈T,sp〉)2:         complement=1.0▹ Initialize product of complements3:          **for all** tr in T **do**4:                 $env_{tr}=\langle tr,sp\rangle$5:                 $V_{env_{tr}}=\mathrm{Execute}\text{-}\sin\mathrm{gle}\text{-}\mathrm{attack}\text{-}\mathrm{propagation}\text{-}\mathrm{trials}\left(numOfTrials,env_{tr}\right)$6:                 $pai_{tr}=\mathrm{Sin}\mathrm{gle}\text{-}\mathrm{attack}\text{-}\mathrm{trace}\text{-}\mathrm{pai}\left(V_{env_{tr}}\right)$7:                 $complement=complement\times \left(1-pai_{tr}\right)$▹ Update product of complements8:         p=1−complement▹ Compute complement of product of complements9:         **return** *p*10: **procedure** Single-attack-trace-pai($V_{env}$)11:         $n\;=\;|V_{env}|$▹ Size of vector of trial outcomes12:         sum=013:         **for** j=1 to *n* **do**14:                 $sum=sum+v_{j}$▹ Update sum of trial outcomes15:         $p=\frac{sum}{n}$▹ Compute expected probability16:         **return** *p*

In Algorithm 2, procedure MULTI-ATTACK-TRACE-PAI$\left(numOfTrials,\mathrm{env}=\langle T,sp\rangle \right)$ computes the cumulative probability of attack impact over the set of multiple attack traces T given the sensor placement configuration sp and the number of MC trials numOfTrials to execute for each attack trace. The procedure calls the EXECUTE-SINGLE-ATTACK-PROPAGATION-TRIALS procedure from Algorithm 1 to compute the vector of trial outcomes for each attack trace and then calls procedure SINGLE-ATTACK-TRACE-PAI to compute the expected probability of attack impact for each trace. In procedure MULTI-ATTACK-TRACE-PAI, the overall probability of attack impact due to any attack trace in T is computed by Equation (8) as the complement of a product of complements.

In the following section, we discuss the extensibility features of the proposed model, including how it may be used to capture customized threats, vulnerabilities, and defensive sensor analytics.

### 4.4. Cybersecurity Sensor Placement Risk Model Extensibility

As described in Section 4, the proposed cybersecurity sensor placement risk model is intentionally generalized for use across a wide variety of network and cyber–physical environments and against a wide variety of cyber attack threats. The model has a modular design in which different sub-models focus on different aspects and dynamics of the COSP problem, and these sub-models combine to capture the full problem and compute the overall risk assessment. The modular design is also intentional and promotes model extensibility and customization. For example, suppose a particular COSP problem instance includes some sensors that have been tested for attack detection performance and other sensors for which test data is not available. In this case, the proposed risk model can be extended to leverage the performance results of tested sensors. Because the sensor detection sub-model (Section 4.2) is a stand-alone model whose dynamics are not interwoven with other COSP-related dynamics that occur at different scales and capture different problem aspects, it is straightforward to substitute sensor performance results for sensors that have been tested while still employing the generalized sensor detection sub-model to capture detection performance for other untested sensors.

Similarly, if there exists a custom threat model that captures the dynamics of a particular attack technique’s process to exploit a particular vulnerability, then this custom threat model can be used to capture the probability of compromise when an attack step that uses the technique against a system component that contains the targeted vulnerability is to be modeled, and still employ the component-vulnerability-to-threat sub-model (Section 4.1) for other attack steps which use other techniques to exploit other vulnerabilities.

Finally, the modular model design enables capture of *zero-day* effects, such as zero-day vulnerabilities and/or exploits, that is, vulnerabilities and exploits that have not been observed or recorded in public sources but are hypothesized to exist. Although the proposed model is not explicitly designed to capture fine-grained details related to zero-day effects, it can, in its present form, provide a coarse-grained capture of such effects. When a COSP problem instance includes hypothesized zero-day threats, corresponding sensor placement risk sub-models can be customized to capture associated zero-day effects. For example, when a zero-day vulnerability is hypothesized to exist on a given system component, the component-to-threat sub-model can be customized to capture this unknown vulnerability by assigning the maximum value to the vulnerability’s CVSS score in Equation (5). This setting represents a vulnerability of maximal severity, and thus the sub-model computes a probability of compromise of 1.0, which one would expect when a zero-day vulnerability is attacked. When a zero-day exploit is to be modeled, the sensor detection sub-model can be customized to capture the sensor’s reduced probability of detecting the exploit by assigning an appropriately adjusted value to variable $dp\left(s,as_{i},t_{j}\right)$ from Equation (6) for an attack step that utilizes the exploit. The adjusted value can be conservatively set as low as 0.0 if desired, representing a worst-case scenario in which the previously unobserved exploit is undetectable by existing sensor analytics. As mentioned above, the proposed model, while able to capture some zero-day details, is not designed to provide fine-grained modeling of zero-day effects. We recognize the development of highly detailed zero-day models as a direction of future work.

## 5. Case Study for Model Demonstration

**Figure 2:**
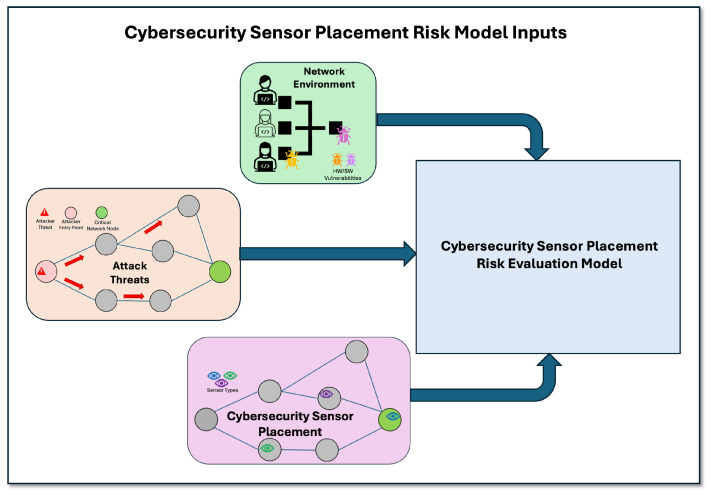
Inputs to the cybersecurity sensor placement risk model include network environment data, attack threat data, and cyber sensor placement data. In this case study, we utilize attack techniques from real cyber breach incidents recorded in the MITRE ATT&CK knowledge base.

To demonstrate the use of the cybersecurity sensor placement risk evaluation model, we conduct a case study on a representative network system for a multi-step attack scenario. Setting up an attack scenario to be evaluated entails specification of model inputs concerning the network environment, attack threats, and sensor defenses. Figure 2 provides a graphical view of model inputs that are used to specify a given attack scenario.

From Figure 2, model inputs include network environment data (green block located at the top of the figure) that specify network devices, interconnections between devices, and hardware and software vulnerabilities present. Model inputs also include the attack threat(s) of concern (orange block located to the left of the figure), which are specified via attack trace(s) (Section 3, Definition 1), and a defensive cybersecurity sensor placement (lavender block located at the bottom of the figure) that aims to protect the network from attack. For this case study, we capture realistic multi-step attacks that employ techniques observed in real cyber breach incidents taken from the MITRE ATT&CK knowledge base. The following describes the network environment and attack scenario captured in this case study.

**Figure 3:**
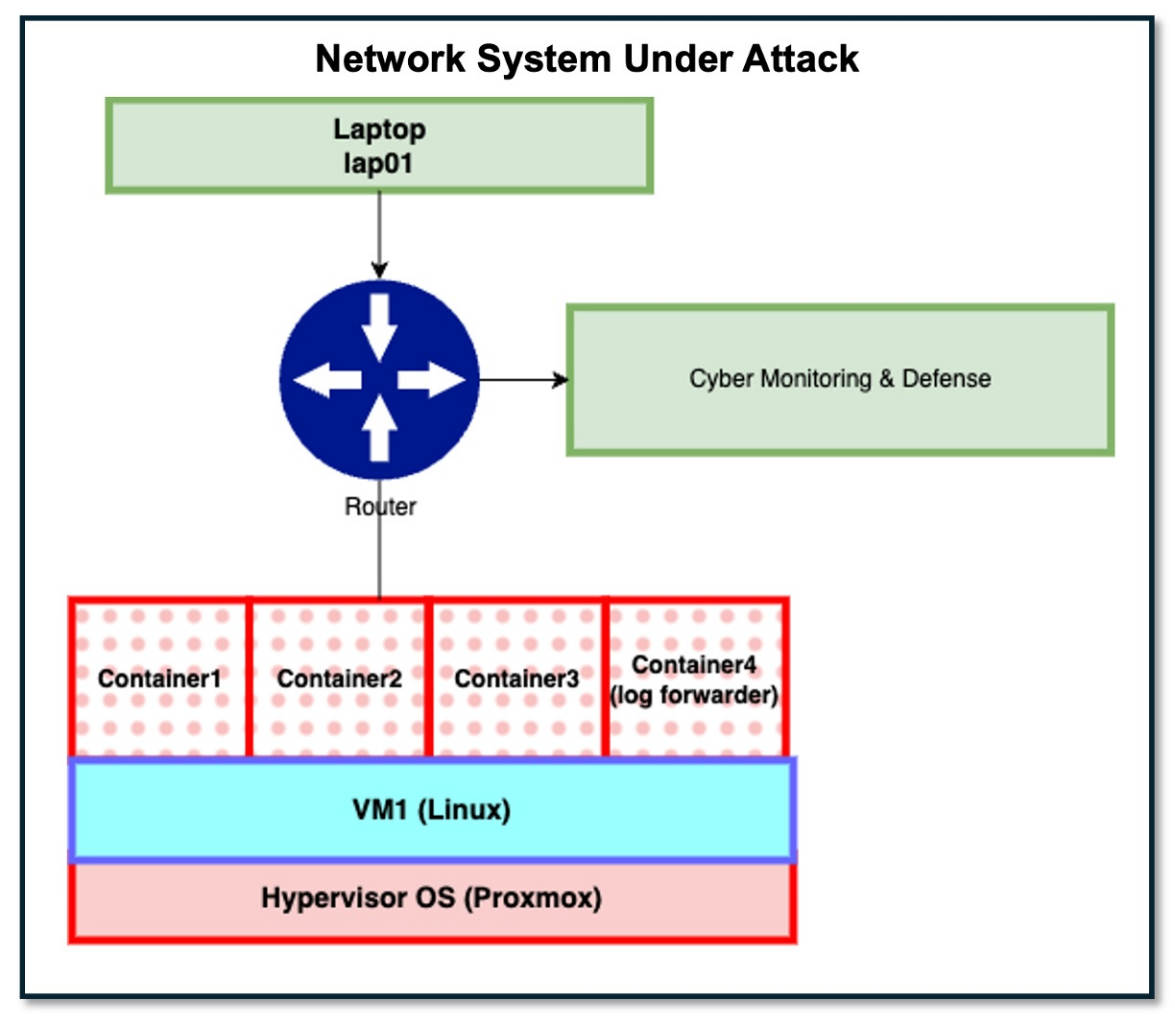
Representative network system with containerized services and infrastructure to support defensive cybersecurity sensors and monitoring.

Consider the network system depicted in Figure 3. The figure captures a system in which users connect to the network with a mobile device, such as a laptop or personal electronic device (e.g., smartphone), and interact with authorized services contained in one or more containers hosted by one or more virtual machines (VMs). The VM(s) are run on a physical host machine and managed by a hypervisor operating system. In the figure for clarity and simplicity, we show just a single laptop and a single VM.

From Figure 3, a user on laptop “lap01” connects to the system and is routed to services contained in containers “Container1”, “Container2”, and/or “Container3”. The network system also includes infrastructure for defensive cybersecurity sensors and monitoring: “Container4” contains services that collect and forward logs generated by cybersecurity data sources to the “Cyber Monitoring and Defense” component, where they are processed by threat detection analytics. Note that “Container4” is intended to contain only services that support defensive sensors and monitoring; it is not intended to contain user-facing services or to be accessed by non-administrative users. For this case study, we assume the laptop runs the Microsoft Windows 10 Pro operating system [84] and all containers run Linux Ubuntu 22+ operating systems [85].

Given the network system described above, we consider an attack threat consisting of two attack traces: one that seeks to disrupt user-facing services and one that seeks to disrupt the system’s cybersecurity sensor and monitoring infrastructure. The attacks described below are intended to capture a realistic multi-step attack that spans multiple system components and leverages a diversity of attack techniques against a modern network environment with a commonly deployed network configuration. We verified the described attack techniques on the network configuration given above by tests executed in a cyber range. The following sections discuss these two traces.

### 5.1. Attack Threat Trace: Disrupt User-Facing Containerized Services

**Figure 4:**
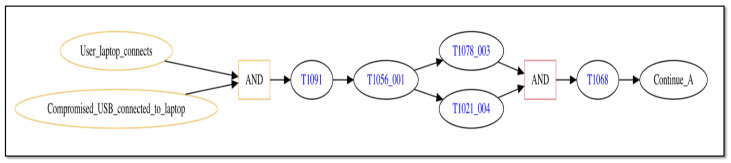
Initial steps of an attack trace intended to disrupt user services.

The first attack trace we consider seeks to disrupt containerized user services. Figure 4 provides a graphical view of the initial attack steps in the trace. The figure depicts the trace as a graph in which nodes represent attack steps or network conditional states and edges represent transitions between a previous step or conditional state to a new step/conditional state.

From Figure 4, the attack trace starts off when a user connects to the network system with a laptop and also connects a compromised USB device to the laptop (the two left-most nodes of the diagram). When both of these conditions occur (immediate successor node labeled as “AND”), the attack is now able to compromise the laptop by taking advantage of Autorun features on removable media that execute upon connection (successor node labeled by MITRE ATT&CK technique “T1091” [86]). The compromised USB installs a key logger application on the laptop, which can intercept user credentials as they type them (successor node labeled by technique “T1056_001” [87]). Using the stolen credentials, the attacker now uses secure file copy ([88]) to copy an exploit file from the laptop to a container with user services (successor node “T1078_003” [89]) and also uses secure shell to connect to the container (node “T1021_004” [90]). Once the attack has executed both of these steps (successor node labeled as “AND”), the exploit file is executed to gain root privileges on the container (node “T1068” [91]). Node “Continue_A” is a marker node that indicates the attack trace continues with additional steps that are shown in the following diagrams.

**Figure 5:**
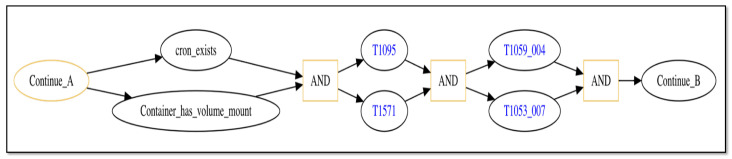
Intermediate steps of an attack trace intended to disrupt user services.

Figure 5 depicts the continuation of the attack trace. If the container has a mounted directory on the host VM (e.g., /var directory) that the user has access to and a cron job ([92]) exists on the mounted directory (nodes labeled as “Container_has_volume_mount” and “cron_exists” followed by “AND” node), then the exploit copies a reverse shell executable ([93]) to the container that will open up a TCP socket connection on a non-standard port (nodes “T1095” [94] and “T1571” [95] followed by “AND” node) to establish a communication channel back to the laptop malware, which runs ncat ([96]) in the socket to enable reading and writing across the channel. The exploit then adds a script to the cron job that executes the reverse shell (rshell) command every minute to receive and send messages repeatedly over the channel (nodes “T1053_007” [97] and “T1059_004” [98] followed by “AND” node). Node “Continue_B” is a marker node indicating additional attack steps that will be shown in the following diagram.

**Figure 6:**
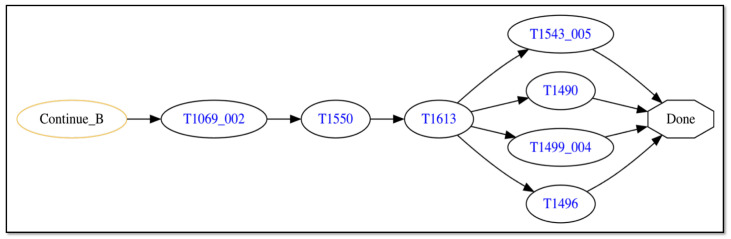
Final steps of an attack trace intended to disrupt user services.

Figure 6 depicts the final steps of the attack trace. The malware discovers the Kubernetes credential file on the host (node “T1069_002” [99]) and uses alternative credentials gleaned from this file (e.g., password hashes or Kerberos tickets) to authenticate to the Kubernetes API server (node “T1550” [100]). With this access to the API server, the malware performs reconnaissance to survey the pods and services available on the server (node “T1613” [101]). Now that the malware has full access to the API server, it can achieve its goal by corrupting available services (node “T1543_005” [102]), deleting files and services to inhibit system recovery (node “T1490” [103]), crashing services to cause a denial of service effect (node “T1499_004” [104]), and hijacking container resources to create and/or delete containers at will (node “T1496” [105]).

The attack trace depicted in the figures contains multiple “AND” logic nodes that have two incoming edges emanating from two attack step nodes. When such a sub-trace is present, this represents attack steps that may be executed in parallel, and the trace may only continue beyond the “AND” node once both attack steps have successfully completed. As discussed in Definition 1, the order in which parallel-executing attack steps finish is not consequential, only that they all finish successfully before the attack trace can progress further. Thus, the attack trace depicted in Figure 4, Figure 5 and Figure 6 represents potentially many logically equivalent traces in which parallel-executing attack steps complete in different sequential orders. For simplicity, we do not display all combinations of equivalent attack step sequences represented by the trace.

### 5.2. Attack Threat Trace: Disrupt Cybersecurity Sensor and Monitoring Infrastructure

The second attack trace we consider is intended to disrupt the defensive cybersecurity sensor and monitoring infrastructure. The attacker’s goal is to compromise services that collect and forward logs generated by the network’s cybersecurity data sources to the “Cyber Monitoring and Defense” network component (depicted by the right-most green rectangle of Figure 3) for processing by threat detection analytics. Recall that these services are contained in “Container4” of our representative network system. The attack leverages the same steps as described above for compromising user-facing services contained in the other containers, with the exception that the malware, when surveying the pods and services available (node “T1613” in Figure 6), specifically targets Container4 and corrupts/denies log forwarding services discovered within.

In the following section, we discuss software vulnerabilities present on system components that are targeted by the techniques in the attack traces described above.

### 5.3. System Vulnerabilities Targeted for Attack

The network system under attack (Figure 3) is assumed to contain vulnerabilities that the attacker attempts to exploit to progress the attack traces towards their intended goals. In this section, we discuss these vulnerabilities and the attack steps that leverage MITRE ATT&CK techniques to attack them. It is important to note that not all attack step techniques given in the attack traces described above require an extant vulnerability to succeed. Many steps employ an attack technique that, in the context of the attack trace it is part of, “lives off the land”, meaning that it makes use of privileges gained by previous successful attack steps to execute and further progress the attack. Table 1 provides a listing of attack step techniques from the traces described in Section 5.1 and Section 5.2 with corresponding details on which vulnerabilities are targeted and why they are targeted. Techniques listed with vulnerabilities targeted as “None” indicate “live off the land” steps that do not require extant vulnerabilities to execute.

In Table 1, note that all techniques except one, technique “T1068”, target no vulnerabilities. This is because the attack steps that leverage these techniques make use of previously gained privileges or pre-existing compromise conditions to execute and progress the attack trace to its next step. Technique “T1068”, however, targets vulnerabilities present in the Linux Ubuntu operating system (OS) running on the container in an attempt to exploit them, escalate privileges, and gain control of the container. For this scenario, we collect relevant Ubuntu vulnerabilities from the National Vulnerability Database (NVD [20]). Table 2 provides a listing of recently discovered Common Vulnerabilities and Exposures (CVEs) collected from NVD for Linux Ubuntu OS versions 22+ with corresponding CVE details, including the date published, the CVSS severity score, the CVSS scoring version used, and where further information about it may be found. In the table, Ubuntu CVEs published in 2023 or later are listed.

In the following section, we discuss the defensive cyber sensors that may be enabled to detect attack threats and alert network defenders.

### 5.4. Defensive Cybersecurity Sensors

To counter the attack threats described in the previous sections, we designate a collection of sensors that are applicable and available for use by network defenders for cybersecurity sensors and monitoring. To designate signature-based sensors, we collected all publicly available signature-based sensor analytics from four widely known cyber detection analytics sources: MITRE’s Cyber Analytics Repository [80], Sigma [81], Elastic SIEM [16], and Splunk [17] and mapped them to the MITRE ATT&CK techniques that they are designed to detect. For each technique present in one of our attack traces, we designate a signature-based sensor as available to monitor and detect that technique if there exists at least one publicly available analytic that maps to it. Recall from Section 3.1 that anomaly-based sensors do not aim to detect previously observed attacks but instead are meant to detect generalized malicious behaviors that deviate from normal, non-malicious use patterns. With this in mind, we designate an anomaly-based sensor as available to monitor and detect an attack step technique if that technique involves interactions between two or more hosts, such as a transfer of data or the copying of a file from one component to the other.

**Figure 7:**
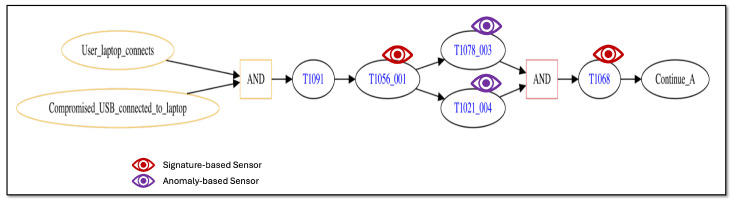
Initial steps of an attack trace intended to disrupt container services with applicable sensors shown.

**Figure 8:**
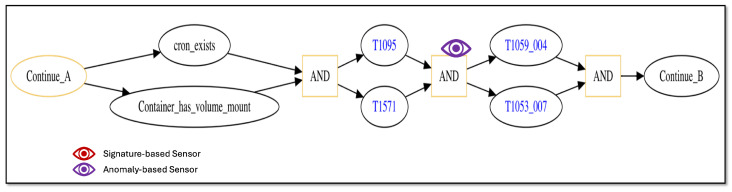
Intermediate steps of an attack trace intended to disrupt container services with applicable sensors shown.

Using this process for our representative scenario, we designate a collection of sensors that can be applied to specific steps in our scenario’s attack traces. Figure 7 and Figure 8 show the initial and intermediate steps of the attack trace described in Section 5.1, respectively, with sensors shown. Note that the final steps of the attack trace shown in Figure 6 are not detectable by either a signature-based or anomaly-based sensor as they all make use of previously gained privileges and execute actions that do not deviate from normal, non-malicious network administration behaviors.

From Figure 7, applicable sensors are depicted by the “eye” graphic placed on the attack step node for the technique that they can monitor, where signature-based sensors are colored red and anomaly-based sensors are colored purple. In the figure, attack steps employing MITRE ATT&CK techniques “T1056_001” and “T1068” (nodes labeled as “T1056_001” and “T1068”, respectively) may be monitored by a signature-based sensor while steps employing techniques “T1078_003” and “T1021_004” (nodes labeled as “T1078_003” and “T1021_004”, respectively) may be monitored by an anomaly-based sensor.

From Figure 8, applicable sensors are depicted in the same way as depicted in Figure 7. In the figure, an anomaly-based sensor may be used to monitor the simultaneous occurrence of attack steps employing techniques “T1095” and “T1571” (shown by the “eye” graphic with purple color on the “AND” node immediately following nodes “T1095” and “T1571”).

The sensors shown in Figure 7 and Figure 8 represent distinct sensors available for use to detect attack step actions. While there are only five sensors shown, each distinct sensor may correspond to multiple sensor instances depending on the network system’s structure. For the representative network system of Figure 3, there are multiple containers, and thus a distinct sensor intended to monitor a container data source may correspond to multiple sensor instances, one for each container present. Table 3 lists all sensor instances available for our representative network system and attack scenario. From the table, there are eight total sensor instances available for use, five signature-based and three anomaly-based. The table also lists the sensor’s data source and the attack technique it monitors.

As discussed in Section 4.2, it is necessary to specify detection probabilities for individual sensor instances. Ideally, data generated from sensor tests or other relevant analyses should be used to specify these. When these are not available, it is necessary to estimate individual detection probabilities. For our case study, we use the estimation method described in Section 4.2 to estimate detection probabilities for signature-based sensor instances. Specifically, we leverage a curated analytics coverage data repository ([112]) that maps each MITRE ATT&CK technique to publicly available signature-based analytics from prevalent sensor analytics sources. With data from this curated mapping, we estimate the detection probability of a given signature-based sensor instance to detect a given attack technique using Equation (6) in which the upper bound of total analytics available is specified from the full data distribution. For anomaly-based sensor instances, since we do not have access to test data, we base the estimate on prior belief with no bias, either positive or negative, and thus we specify detection probability as 0.5. Table 4 lists the eight sensor instances and their corresponding estimated detection probabilities.

In the following section, we describe a second scenario that is a slight variant of the case study scenario given above that includes zero-day effects.

### 5.5. Case Study Scenario Variant: Inclusion of Zero-Day Effects

**Figure 9:**
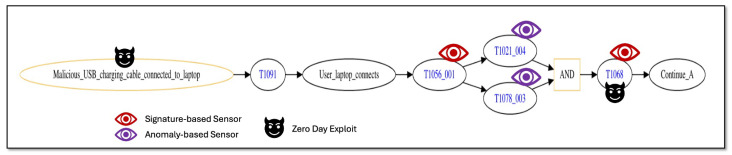
Initial steps of an attack trace intended to disrupt container services that employs zero-day exploits.

In Section 4.4, we described how zero-day effects may be captured by the cybersecurity sensor placement risk evaluation model (Section 4). Here, we construct a second scenario that is a small variation in the scenario of the previous section to illustrate the inclusion of zero-day effects. Figure 9 provides a graphical depiction of the initial steps of the original attack trace of Figure 4 that includes zero-day exploits.

From Figure 9, two zero-day exploits are included in the initial attack trace steps (depicted by the black “devil” icons). Recall from Section 5.1 that the original scenario started with a malicious USB device containing malware connected to the user’s laptop. In that scenario, the assumption is that the USB malware has already gained control of the laptop, and thus when the laptop connects to the network, the attack can immediately progress and attempt further attack steps. In this alternative scenario, the laptop starts out in an uncompromised state, and then the user attempts to charge the laptop with a USB charging cable that includes a zero-day exploit (black “devil” icon to the left of the diagram). The zero-day exploit contained in the charging cable affords a remote attacker connected to the same Wifi network as the laptop the ability to execute malicious code on the laptop in an attempt to compromise it and gain control. This previously unknown USB charging cable exploit was created by a white-hat hacker in 2019 to raise awareness that charging cables, often assumed not to pose a threat, may indeed be leveraged as a viable threat vector for attackers to exploit [113]. The second zero-day exploit (black “devil” icon to the right of Figure 9) leverages a previously unknown exploit to escalate privileges on the container and gain control as root. The remaining attack trace steps and defensive sensors for the second scenario are the same as described for the first scenario (Figure 5, Figure 6, and Figure 8).

Table 5 provides a listing of attack step techniques for the second scenario with corresponding details on which vulnerabilities are targeted and why they are targeted. Similar to Table 1, techniques listed with vulnerabilities targeted as “None” indicate “live off the land” steps that do not require extant vulnerabilities to execute. From the table, all rows are the same as given in Table 1 with the exception of the first row (MITRE ATT&CK Technique “T1091”) and the fifth row (technique “T1068”). Windows 10 vulnerabilities on the laptop are targeted by the first zero-day exploit (row with technique “T1091”) corresponding to the USB charging cable exploit. As in the first scenario, technique “T1068” targets Ubuntu vulnerabilities on the container, but employs a zero-day exploit rather than a previously observed exploit.

For this second scenario, the same Ubuntu vulnerabilities are targeted by the attack step employing technique “T1068” as given for the first scenario in Table 2. This zero-day scenario, however, also contains an attack step that targets Windows 10 vulnerabilities on the laptop. Table 6 lists these Windows 10 vulnerabilities.

For this zero-day scenario, the same eight sensor instances as given in Table 3 are used. However, because a zero-day exploit is employed to execute the attack technique “T1068” on a container, signature-based sensor instances that monitor container data sources for that technique (sensor instances #4–#7 of Table 3) are assumed to have lower than normal detection accuracy. Thus, an adjusted lower value for the detection probability should be specified based on prior belief of confidence and/or stakeholder risk tolerance levels. As discussed in Section 4.4, a worst-case situation can be captured by specifying a detection probability of 0.0, signifying that the previously unobserved exploit is undetectable by existing signature-based sensor analytics. Table 4 lists the estimated detection probabilities for sensor instances of the first (non-zero-day) scenario; note that the affected sensor instances (instances #4–#7 in the table) have an estimated detection probability of 0.45. For this zero-day scenario, we assume degraded but not completely decimated sensor performance, and thus specify a detection probability of 0.225 for affected sensor instances, which represents a performance degradation of 50%.

## 6. Experiments

The case study given in Section 5 describes two experimental scenarios, one that does not include zero-day effects and one that does include such effects. Each scenario includes a total of four attack traces, one trace corresponding to each of the four containers in the network system environment depicted in Figure 3. In this section, we discuss experiments conducted on these two scenarios. We refer to the scenario without zero-day effects as “Scenario 1” and the scenario with zero-day effects as “Scenario 2”.

As discussed in Section 5.4 and shown in Table 3, there are a total of eight sensor instances that may be used in any combination to detect attacks. With these sensor instances, there is a total of $2^{8}=256$ sensor placements that may be deployed, including a placement in which no sensor instances are used. We examine a sampling of possible sensor placement configurations that vary the number of instances deployed and capture a range of sensor placements that spans from minimal or no sensor use to maximal sensor use (i.e., deploying all possible sensor instances). Table 7 gives the sensor placement configurations tested for both experimental scenarios.

**Figure 10:**
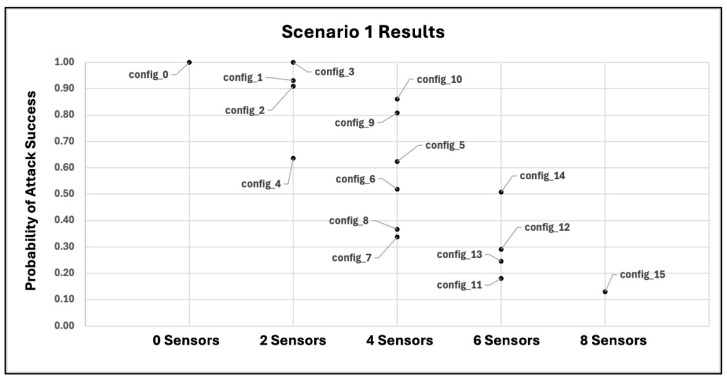
Experimental Results for Scenario 1—Attacks that do not include zero-day exploits.

**Figure 11:**
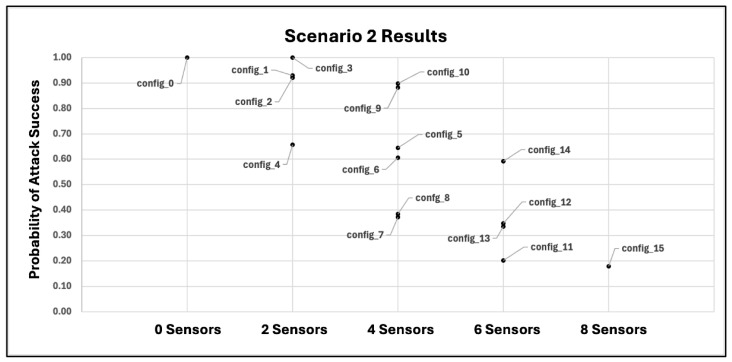
Experimental Results for Scenario 2—Attacks that include zero-day exploits.

A set of $10^{4}$ MC trials on the cybersecurity sensor placement risk model (Section 4) is executed for each of the sensor placement configurations in Table 7, and individual trial results are aggregated to compute the expected probability of attack success given the sensor placement deployed. Figure 10 and Figure 11 provide a graphical representation of the experimental results for Scenarios 1 and 2, respectively.

In Figure 10 and Figure 11, the horizontal axis groups results by the total number of sensor instances used, while the vertical axis measures the expected probability of attack success when a given sensor placement configuration is deployed. Plotted points represent results for individual sensor placements that correspond to configurations given in Table 7 and are labeled by their sensor placement configuration ID from that table.

From Figure 10 and Figure 11, “config_0”, representing a sensor placement configuration that uses no sensor instances, yields a probability of attack success of 1.0 as expected while “config_15”, which represents a sensor placement configuration with maximal sensor use (all eight sensor instances), yields the best (lowest) probability of attack success for both experiments, 0.13 and 0.18 for Scenarios 1 and 2, respectively. Because results for configuration “config_15” represent the best risk mitigation possible via a sensor-based defense, network administrators may wish to consider additional non-sensor-based cybersecurity controls to mitigate the remaining risk and provide “defense-in-depth” as is recommended by the National Institute of Standards and Technology (NIST) in the Cyber Risk Management Framework (RMF) [120]. Results from both experiments also exhibit a general trend of greater security (less risk) as more sensor instances are deployed, which is also expected.

However, as can be observed in Figure 10 and Figure 11, some sensor placement configurations outperform others *even though they deploy a smaller number of sensor instances*. This is an important result that underscores the value of the cybersecurity sensor placement risk model: some combinations of sensor instances are critical to the network’s security posture in the presence of the given attack threats, while other combinations are much less impactful despite deploying a larger number of sensor instances. The proposed model provides network administrators a tool to quickly and quantitatively compare different sensor placement configurations for defensive performance and discover combinations of sensor instances that provide the best “bang for the buck”, that is, the most defensive benefit for the least sensor resource cost.

For the experimental results given in Figure 10 and Figure 11, we quantify confidence by computing MC standard errors and generating 95% confidence intervals [121,122]. The errors and confidence intervals associated with the results of Figure 10 and Figure 11 are given in Table 8 and Table 9, respectively.

From Table 8 and Table 9, the first and second columns give the sensor placement configuration ID and expected probability of attack success as is graphically shown in Figure 10 and Figure 11, respectively, while the third and fourth columns give the associated MC standard errors and confidence intervals with 95% confidence level. As can be seen from the tables, errors are relatively small and confidence intervals show relatively tight bounds, indicating that the experimental set size of $10^{4}$ MC trials is sufficient to estimate the actual expected probability of attack success with relatively high confidence.

## 7. Discussion of Practical Considerations, Model Limitations, and Future Work

In this study, we propose an extensible mathematical model for defensive cybersecurity sensor placement evaluation that considers both the sensor data source locations and the sensor analytics/rules used, the combination of which has not been studied previously. The model computes the inherent risk when a given sensor placement configuration is deployed to detect a given set of potentially multi-step attacks. In this section, we discuss practical considerations for applying the model to real-world systems, model limitations, and several avenues of future work to extend/enhance the model and combine it with other algorithmic techniques to support intelligent defensive cybersecurity OSP.

### 7.1. Applying the Model to Real-World Network Systems

We provide a non-exhaustive discussion of practical considerations for applying the proposed sensor placement risk evaluation model to an existing network system. For clarity, we organize the discussion into broad categories of related considerations and describe aspects specific to each category.

#### 7.1.1. Obtain Network System Environment Information

Developing an applicable set of attack threats that can exploit a given network system’s vulnerabilities and achieve the attacker’s goals is an important prerequisite for effectively applying the proposed sensor placement risk model. Because attack threats target the attack surface landscape within the system, having a detailed and accurate description of the system’s attack surfaces, including components, their vulnerabilities, and interconnections between components, is necessary to determine a set of system-relevant attack threats that are to be mitigated. However, obtaining this information for sufficiently complex, larger-scale network systems may face challenges such as incomplete system documentation or a lack of access to system components containing sensitive data. Ensuring that network defenders have the proper access to system component information while also protecting the sensitive data residing on components is thus important. Additionally, network tools such as Nessus [123] and RedSEAL [124] are prevalent examples of useful applications that can facilitate the gathering of network environment information.

#### 7.1.2. Determine Attack Threats of Concern

Even when detailed network system information is readily available, determining attack threats of concern manually can be a time-consuming and resource-intensive process [125]. Automatic generation of attack threats, although challenging in itself, is thus a promising direction of future work [126] that we discuss further in Section 7.3. Despite these challenges, significant benefits can still be gained by developing, either manually or through some automated means, a partial set of attack threats. Applying the proposed model to evaluate the inherent risk posed by even an incomplete set of attack threats can serve to identify gaps in sensor-based defenses that otherwise might not be known.

#### 7.1.3. Specify Sensor Detection Rates/Probabilities

To evaluate a given cybersecurity sensor placement against a given set of attack threats, it is necessary to estimate sensor detection probabilities for available sensor instances against attack threats of concern. As discussed in Section 4.2, ideally, detection rates are specified via data from test results, red team exercises, or other relevant analyses. When such data is not available, we propose the generalized estimation method described in Section 4.2 and given by Equation (6). However, for a specific network system, building a reliable, foundational dataset of sensor detection performance through experimentation and analyses remains important, and ultimately serves to increase the accuracy of sensor placement risk evaluations.

#### 7.1.4. Model Complexity and Scalability

In this section, we provide a formal analysis of the computational complexity of the proposed model and discuss model scalability with respect to model runtimes. As described in Section 4, the full model is composed of four sub-models, namely the component vulnerability-to-threat sub-model, the sensor detection sub-model, the single attack propagation sub-model, and the multi-attack aggregation sub-model. Here, we discuss the complexity of each sub-model and the full model as a whole.

We start with the component vulnerability-to-threat sub-model. As described in Section 4.1 and specified by Equation (4), this sub-model computes the probability of compromise $p_{compromise}\left(cmp\right)$ when an attack is initiated on a given component cmp with a given vulnerability profile *V*. If |V| represents the number of vulnerabilities in *V*, then the computational complexity of $p_{compromise}\left(cmp\right)$ is given by

(9) $$C_{p_{compromise}\left(cmp\right)}=O\left(|V|\right),$$

where $C_{p_{compromise}\left(cmp\right)}$ represents the complexity of $p_{compromise}\left(cmp\right)$ and *O* represents the order of approximation notation. From the equation, complexity is on the order of |V| because $p_{compromise}\left(cmp\right)$ is computed by executing the scalar function given in Equation (5) for each vulnerability v∈V.

As described in Section 4.2, the sensor detection sub-model is given by Equation (6), which makes use of a pre-computed mapping of attack techniques to available detection analytics from the MITRE ATT&CK knowledge base. Specifying the detection probabilities for a collection of sensors involves a one-time computational cost that is O(k), where *k* represents a constant.

The single attack propagation sub-model described in Section 4.3.1 and given by Algorithm 1 computes $p_{compromise}\left(cmp\right)$ for each attack step $as_{i}$ in the attack sequence AS of a given attack trace tr. If n=|AS| represents the number of attack steps in AS, then the computational complexity incurred by executing a single MC trial on tr is given by

(10) $$C_{tr_{oneTrial}}=\sum_{i=1}^{n}\left|V_{i}\right|,$$

where $V_{i}$ is the set of vulnerabilities on the component attacked by attack step $as_{i}$, $|V_{i}|$ is the number of vulnerabilities in $V_{i}$, and $C_{tr_{oneTrial}}$ represents the complexity of executing one trial on attack trace tr. If $V_{max}$ represents the largest set of vulnerabilities on any attacked component, that is $|V_{max}\left|\;\geq \;\right|V_{j}|,\forall V_{j}\;j=1,\ldots ,n$, then $C_{tr_{oneTrial}}=O\left(\left|AS|\;\times \;\right|V_{max}|\right)$. The complexity of executing a set of multiple MC trials on attack trace tr is thus given by

(11) $$C_{tr_{multiTrial}}=O\left(|MC_{trials}\left|\;\times \;|AS|\;\times \;\right|V_{max}|\right),$$

where $|MC_{trials}|$ represents the size of the set of MC trials executed and $C_{tr_{multiTrial}}$ is the overall complexity associated with the single attack propagation sub-model.

As discussed in Section 4.3.2, the multi-attack aggregation sub-model links all other sub-models to compute the risk function of Equation (1), representing the overall risk value for a given sensor placement assessment. As specified in Algorithm 2, the sub-model executes the single attack propagation sub-model for each attack trace tr in the set of attack traces to be evaluated T. If $AS_{max}$ represents the attack sequence of an attack trace with the largest number of attack steps, that is, $|AS_{max}|\;\geq \;|AS_{j}|,\forall AS_{j}\;j=1,\ldots ,\left|T\right|$, then complexity is given by

(12) $$C_{T}=O\left(|T|\;\times \;|MC_{trials}|\;\times \;|AS_{max}\left|\;\times \;\right|V_{max}|\right),$$

where |T| is the number of traces in T and $C_{T}$ is the complexity associated with the multi-attack aggregation sub-model and represents the overall complexity of the full model.

As can be seen from Equation (12), complexity depends on the number of attack traces evaluated, the maximum number of attack steps contained in any attack trace, the maximum number of vulnerabilities on any attacked component, and the number of MC trials executed. For experiments in which the number of MC trials is much greater than the other factors, that is, $|MC_{trials}|\;>>\;|T|,|AS_{max}\left|,\right|V_{max}|$, then the overall complexity is dominated by how many MC trials are executed.

The model is implemented in Python version 3.9.0 [127] and the experiments given in Section 6 are conducted using a Macbook Pro laptop manufactured by Apple, Inc., Cupertino, CA, USA, with an Apple M3 Pro chip and 36 GB of memory. Each experiment executes a set of $10^{4}$ MC trials, and model runtimes were approximately one to two seconds per experiment. Preliminary experiments conducted on larger problem instances and for trial sets of $10^{5}$ and $10^{6}$ MC trials exhibit runtimes of approximately three to five seconds. Based on these results, we estimate that, for scenarios in which the number of MC trials executed is the dominant factor, upper bound model runtimes for large-scale problem instances are on the order of minutes.

### 7.2. Model Limitations

In this section, we discuss model limitations, and in the following section, we describe several directions for future work that can address these limitations. As discussed in Section 7.1, applying the proposed model in real-world settings entails obtaining network system environment data and determining attack threats of concern, both of which may be challenging tasks for sufficiently complex network systems. While some tools exist and others are being developed to ameliorate these challenges, it is often not feasible to have perfect knowledge of all system vulnerabilities and relevant exploits. When this is the case, risk evaluations generated by the model under incomplete data will be relatively less accurate than if perfect vulnerability and exploit data are available. As mentioned in the previous section, there are significant benefits to be gained by applying the model to generate risk evaluations even under incomplete information. The model provides an empirically driven estimation of cybersecurity risk that serves to identify gaps in sensor-based defenses and supports the discovery of more effective sensor placements to address gaps by enabling network administrators to estimate and compare risk for multiple alternative sensor placements.

As discussed in Section 4.2, sensor detection probabilities ideally should be set via test results, data collected from red team exercises, or other relevant analyses. However, collecting such data for all available sensors against all attack techniques of concern can be a resource-intensive task. When such data is not available, the model includes a generalized sensor detection probability estimation component for signature-based sensors, which leverages publicly available analytics data repositories that map known sensor analytics to the MITRE ATT&CK techniques they are designed to detect. While this generalized estimation method provides a rough estimation of a sensor’s ability to detect an applicable threat, it does not take into account potential bias present in analytics repositories and focuses primarily on signature-based sensors and not anomaly-based sensors. Additionally, sensor detection probability estimation for zero-day scenarios, which aim to estimate an anomaly-based sensor’s performance degradation in the presence of a zero-day exploit, relies on subjective judgment when empirical sensor test data is unavailable. Thus, a key direction for model enhancement is the development of a curated knowledge base of sensor detection test data relevant to the network environment of concern. This is discussed further in the following section.

In this paper, we demonstrate the proposed model via a case study on a representative network system subject to multi-step attacks taken from the MITRE ATT&CK knowledge base and known vulnerabilities listed in the NVD. It is important to note that while MITRE ATT&CK is updated regularly, there is some lag between when an attack technique is discovered and when the knowledge base is updated. NVD faces a similar lag time between vulnerability discovery and database update. However, the proposed model does not require exclusive use of MITRE ATT&CK and NVD as its source of attack techniques and vulnerabilities; they were only used here to demonstrate the model. Practical applications can use additional sources for specifying attack techniques and vulnerabilities as desired.

### 7.3. Future Work Directions

We outline several directions for future work in the cybersecurity optimal sensor placement research area. For clarity, we organize the discussion into broad categories of related future work efforts.

*Model Extension to Capture Defense-in-Depth*—The proposed cybersecurity sensor placement risk model can be extended to capture a combination of multiple defensive measures that are intended to provide “defense-in-depth” as recommended in the NIST RMF ([120]). Sub-models that capture other, non-sensor-based network hardening security controls, especially those that are needed to implement a zero-trust cybersecurity paradigm, can be developed and incorporated into the sensor placement risk model. Exemplar zero-trust hardening controls that might be modeled include multi-factor authentication, network segmentation and/or micro-segmentation, application whitelisting, patch management policy, and security configuration baselines and monitoring, among others [128]. Because the model is designed for extensibility, a new sub-model capturing a particular security control can be developed and readily incorporated into the larger sensor placement risk model by aggregating its outputs and using these aggregated outputs to adjust the probability of compromise for individual system components generated by the component-vulnerability-to-threat sub-model (Section 4.1).

*Model Extension to Capture After-detection Response*—The sensor placement risk model given in this paper captures defensive performance up to the detection point, but does not model mitigation response after detection has occurred. The proposed model can be extended to capture response to a detected threat, and may consider aspects of response timeliness and completeness, such as delayed response and/or partial threat mitigation.

*Model Extension to Capture Cost*—The proposed model focuses on sensor placement risk and does not address sensor deployment cost. Models may be developed to capture system constraints on sensor analytic processing speed and/or capacity, as well as resource costs for instantiating or modifying sensor deployments.

*Model Enhancement via Improved Sensor Detection Test and Analysis Data*—The proposed model relies on publicly available sources of sensor detection analytics and curated knowledge bases that map sensor analytics to the MITRE ATT&CK techniques they are intended to detect. Despite the utility of such sources, there is much work that can be carried out to extend, enhance, and continuously update the pool of publicly available cybersecurity sensor detection test and analysis data. For example, current knowledge bases address signature-based sensor analytics but do not address anomaly-based analytics. New test data and corresponding analysis and/or knowledge bases can be built, and existing knowledge bases can be continuously updated as new analytics and attack techniques are discovered. Test and analysis data may also seek to incorporate results on both true and false positive rates, as well as sensor analytic processing costs. Richer data sources related to testing and analysis of sensor detection rates would enable more accurate modeling of cybersecurity sensor placement risk and cost.

*Model Extension via Machine Learning Prediction of Sensor Detection Performance*—In addition to improving the pool of available sensor detection test data discussed above, the proposed model can be enhanced by the development of machine learning models to predict sensor analytic accuracy and performance, including predicted rates for true/false positives and true/false negatives. Sufficiently trained prediction models may be used in lieu of actual test data when such data is not available, or may be used to generate synthetic data to augment existing data pools.

*Model Extension to Capture Mission Impact*—The proposed model evaluates risk to a given network system based on its cybersecurity sensor defense, but does not capture risk to the mission that the system supports. Mission impact analysis requires mission decomposition and its mapping to system-level functions, which is frequently a tedious manual process [129]. Development of automated and semi-automated mission-impact models that analyze the performance of a given cybersecurity sensor defense against a given set of attack threats would be a great boon to the field.

*Model Extension to Capture Fine-grained Details of Zero-Day Effects*—The proposed model, while not explicitly designed for zero-day modeling, can be customized in its present form to capture coarse-grained effects related to zero-day threats. Future work can focus on leveraging existing zero-day threat models or developing new models to be used in combination with the proposed sensor placement risk model to capture fine-grained zero-day dynamics and effects.

*Improved Model Ease of Use via Automated/Semi-automated Attack Trace Generation*—The proposed model requires specification of relevant attack threats of concern in the form of a set of attack traces (Section 3.2). The current practice among network security administrators is to do this manually, which, for a sufficiently sized network system, is a tedious and resource-intensive procedure [125]. Leveraging existing automated and semi-automated methods for the generation of cyber attacks and/or the development of new methods would relieve this burden and improve model usability, especially for dynamic network environments in which threats are constantly shifting and evolving.

*Improved Model Ease of Use via Incremental Risk Scenario Change Modeling*—In a real-world setting, a given risk scenario representing the current network environment may be subject to incremental changes such as updated sets of system vulnerabilities (e.g., when patches are deployed or new vulnerabilities are discovered), attack traces (e.g., when new attacks are identified), or detection analytics (e.g., when existing analytics are improved or new analytics are acquired). Frequent and repeated re-specification and re-execution of the proposed model over multiple incremental changes to the network environment is burdensome to model users. Model usability could be improved by the creation of a supplementary “risk change” model that, given a base scenario, its model-generated risk estimate, and a set of incremental changes to scenario inputs, provides a quick-turn coarse estimate of the change in risk relative to the base scenario. The supplementary model could be used in tandem with the proposed full risk model to balance tradeoffs between model accuracy and cost, where the full model is used to generate risk estimates with relatively higher accuracy and higher cost occasionally, while the supplementary model is used to generate risk estimates with relatively lower accuracy and lower cost more often.

*Support for Intelligent and Adaptive Defensive Cybersecurity OSP*—The proposed sensor placement risk model evaluates the defensive performance of a given cybersecurity sensor placement configuration against a given set of attack threats. The model can be paired with an AI-based optimization method to build an intelligent decision support system that searches the space of possible sensor configurations and recommends optimal configurations that maximize security and minimize sensor resource cost. Similar systems have been developed that pair cybersecurity risk models with AI-based optimization methods to construct intelligent adaptive defensive systems for other problems in the cybersecurity domain [15,79,130,131,132]. In such systems, the cybersecurity risk model serves to evaluate candidate defensive solutions generated by an AI-based optimization component, which utilizes these evaluations to explore the space of possible solutions and discover optimal defenses that balance between defensive performance and cost.

## 8. Conclusions

This paper presents an extensible mathematical model for cybersecurity sensor placement risk evaluation that captures both sensor data source locations and sensor analytics/rules used, the combination of which has not been previously studied. The proposed model provides a quantitative evaluation of a given defensive sensor placement against a given set of attack threats for its defensive performance, that is, how likely it is to detect the threats it is intended to detect. This paper also provides a novel and detailed problem formulation of the defensive cybersecurity optimal sensor placement problem that serves to clearly specify the relevant details of sensor-based cyber defense. Model usage is demonstrated via a detailed case study on a representative network system under threat of multi-step cyber attacks that employ real attack techniques taken from the MITRE ATT&CK knowledge base. Additionally, this paper provides a discussion outlining several key avenues of future work that may be pursued to progress the field of cybersecurity optimal sensor placement research. The aim is to support the adaptation of techniques and methods developed for OSP in other problem domains to the cybersecurity domain.

## Figures and Tables

**Table 1:** Attack step techniques and their targeted vulnerabilities.

MITRE ATT&CK Technique	Vulnerabilities Targeted	Reason
T1091	None	Laptop already compromised by malware on connected USB
T1056_001	None	Installed key logger steals credentials
T1078_003	None	Copy file to container allowed after login
T1021_004	None	ssh to container allowed after login
T1068	Ubuntu vulnerabilities	Escalation of privilege exploit attempt on container
T1095	None	Communication via non-application layer protocol (TCP) enabled by privlege escalation
T1571	None	Non-standard port use allowed after login
T1053_007	None	cron script modification enabled by privilege escalation
T1059_004	None	cron job execution allowed in normal container environment
T1069_002	None	Discovery of Kubernetes credential file enabled by privilege escalation
T1550	None	Access to credentials file enabled by privilege escalation
T1613	None	Discovery of container services enabled by privilege escalation
T1543_005	None	Modification of service enabled by privilege escalation
T1490	None	Deletion of service enabled by privilege escalation
T1499_004	None	Crashing of service enabled by privilege escalation
T1496	None	Deletion of service-providing containers and/or creation of non-useful containers enabled by privilege escalation

**Table 2:** Targeted Linux Ubuntu OS CVEs with corresponding details.

CVE	Date Published	CVSS Score	CVSS Version	Reference
CVE-2024-5290	7 August 2024	7.8	3.1	[106]
CVE-2024-1724	25 July 2024	8.2	3.1	[107]
CVE-2023-5536	11 December 2023	6.4	3.1	[108]
CVE-2023-32629	25 July 2023	7.8	3.1	[109]
CVE-2023-2640	25 July 2023	7.8	3.1	[110]
CVE-2023-30549	25 April 2023	7.8	3.1	[111]

**Table 3:** Cybersecurity sensor instances available for use to detect attack step actions.

Sensor #	Sensor Type	Sensor Data Source	Technique Monitored
1	Signature-based	Laptop (lap01)	T1056_001
2	Anomaly-based	Network communication from laptop (lap01) to container	T1078_003
3	Anomaly-based	Network communication from laptop (lap01) to container	T1021_004
4	Signature-based	Container1	T1068
5	Signature-based	Container2	T1068
6	Signature-based	Container3	T1068
7	Signature-based	Container4	T1068
8	Anomaly-based	Network communication from laptop (lap01) to container	T1095 AND T1571

**Table 4:** Estimated detection probabilities for sensor instances of Table 3.

Sensor #	Sensor Type	Detection Probability Estimate
1	Signature-based	0.02
2	Anomaly-based	0.5
3	Anomaly-based	0.5
4	Signature-based	0.45
5	Signature-based	0.45
6	Signature-based	0.45
7	Signature-based	0.45
8	Anomaly-based	0.5

**Table 5:** Zero-day attack scenario: Attack step techniques and their targeted vulnerabilities.

MITRE ATT&CK Technique	Vulnerabilities Targeted	Reason
T1091	Windows 10 vulnerabilities	Attempt to install malware onto laptop by malicious USB charging cable (zero-day exploit)
T1056_001	None	Installed key logger steals credentials
T1078_003	None	Copy file to container allowed after login
T1021_004	None	ssh to container allowed after login
T1068	Ubuntu vulnerabilities	Escalation of privilege exploit attempt on container (zero-day exploit)
T1095	None	Communication via non-application layer protocol (TCP) enabled by privlege escalation
T1571	None	Non-standard port use allowed after login
T1053_007	None	cron script modification enabled by privilege escalation
T1059_004	None	cron job execution allowed in normal container environment
T1069_002	None	Discovery of Kubernetes credential file enabled by privilege escalation
T1550	None	Access to credentials file enabled by privilege escalation
T1613	None	Discovery of container services enabled by privilege escalation
T1543_005	None	Modification of service enabled by privilege escalation
T1490	None	Deletion of service enabled by privilege escalation
T1499_004	None	Crashing of service enabled by privilege escalation
T1496	None	Deletion of service-providing containers and/or creation of non-useful containers enabled by privilege escalation

**Table 6:** Targeted Windows 10 OS CVEs with corresponding details.

CVE	Date Published	CVSS Score	CVSS Version	Reference
CVE-2023-6080	18 October 2024	7.8	3.1	[114]
CVE-2023-7016	27 February 2024	7.8	3.1	[115]
CVE-2023-5993	11 December 2024	7.8	3.1	[116]
CVE-2023-32544	19 January 2024	5.5	3.1	[117]
CVE-2023-29244	19 January 2024	7.8	3.1	[118]
CVE-2023-47145	7 January 2024	7.8	3.1	[119]

**Table 7:** Sensor placement configurations tested for both experimental scenarios. Each configuration includes a combination of sensor instances from Table 3. In column “Sensor Types”, the number of sensors of each type is shown. Signature-based sensors are listed as “Sig.”. Anomaly-based sensors are listed as “Anom.”. For data source locations and techniques monitored by individual sensor instances, please see Table 3.

Sensor Placement Config. ID	Sensor Instances	Sensor Types
config_0	None	None
config_1	Sensors #1–#2	1 Sig., 1 Anom.
config_2	Sensors #3–#4	1 Sig., 1 Anom.
config_3	Sensors #5–#6	2 Sig.
config_4	Sensors #7–#8	1 Sig., 1 Anom.
config_5	Sensors #1–#4	2 Sig., 2 Anom.
config_6	Sensors #5–#8	3 Sig., 1 Anom.
config_7	Sensors #3,#4,#7,#8	2 Sig., 2 Anom.
config_8	Sensors #1,#2,#7,#8	2 Sig., 2 Anom.
config_9	Sensors #3–#6	3 Sig., 1 Anom.
config_10	Sensors #1,#2,#5,#6	3 Sig., 1 Anom.
config_11	Sensors #1–#4,#7,#8	3 Sig., 3 Anom.
config_12	Sensors #1,#2,#5–#8	4 Sig., 2 Anom.
config_13	Sensors #3–#8	4 Sig., 2 Anom.
config_14	Sensors #1–#6	4 Sig., 2 Anom.
config_15	Sensors #1–#8	5 Sig., 3 Anom.

**Table 8:** Standard errors and confidence intervals for the experimental results of Scenario 1 shown in Figure 10.

Sensor Placement Configuration ID	Probability of Attack Success	Std. Error	95% Confidence Interval
config_0	1.00	0.0000	[1.000, 1.000]
config_1	0.93	0.0026	[0.925, 0.935]
config_2	0.91	0.0029	[0.904, 0.916]
config_3	1.00	0.0000	[1.000, 1.000]
config_4	0.64	0.0048	[0.631, 0.649]
config_5	0.62	0.0049	[0.610, 0.630]
config_6	0.52	0.0050	[0.510, 0.530]
config_7	0.34	0.0047	[0.331, 0.349]
config_8	0.37	0.0048	[0.361, 0.379]
config_9	0.81	0.0039	[0.802, 0.818]
config_10	0.86	0.0035	[0.853, 0.867]
config_11	0.18	0.0038	[0.172, 0.188]
config_12	0.29	0.0045	[0.281, 0.299]
config_13	0.25	0.0043	[0.242, 0.258]
config_14	0.51	0.0050	[0.500, 0.520]
config_15	0.13	0.0034	[0.123, 0.137]

**Table 9:** Standard errors and confidence intervals for the experimental results of Scenario 2 shown in Figure 11.

Sensor Placement Configuration ID	Probability of Attack Success	Std. Error	95% Confidence Interval
config_0	1.00	0.0000	[1.000, 1.000]
config_1	0.93	0.0026	[0.925, 0.935]
config_2	0.92	0.0027	[0.915, 0.925]
config_3	1.00	0.0000	[1.000, 1.000]
config_4	0.66	0.0047	[0.651, 0.669]
config_5	0.64	0.0048	[0.631, 0.649]
config_6	0.61	0.0049	[0.600, 0.620]
config_7	0.37	0.0048	[0.361, 0.379]
config_8	0.39	0.0049	[0.380, 0.400]
config_9	0.88	0.0033	[0.874, 0.886]
config_10	0.90	0.0030	[0.894, 0.906]
config_11	0.20	0.0040	[0.192, 0.208]
config_12	0.35	0.0048	[0.341, 0.359]
config_13	0.33	0.0047	[0.321, 0.339]
config_14	0.59	0.0049	[0.580, 0.600]
config_15	0.18	0.0038	[0.172, 0.188]

## Data Availability

Data are contained within the article.
